# Cooperative Mineralisation of the Fragrance Ingredient 2‐Cyclohexylidene‐2‐Phenylacetonitrile by a Consortium of a *Variovorax* and an *Acidovorax* Strain Isolated From Activated Sludge

**DOI:** 10.1111/1758-2229.70257

**Published:** 2026-02-04

**Authors:** Tina Haupt, Arturo Mendoza, Yumiko Weiner‐Sekiya, Karen Jenner, Georg Kreutzer, Andreas Natsch

**Affiliations:** ^1^ Fragrances S&T, Ingredients Research Givaudan Schweiz AG Kemptthal Switzerland; ^2^ Regulatory Affairs and Product Safety Givaudan Suisse SA Vernier Switzerland

**Keywords:** activated sludge, biodegradation, consortium, cooperative mineralisation

## Abstract

To be considered ultimately biodegradable, a substance needs to reach ≥ 60% mineralisation in screening tests. Ultimate biodegradability can also be studied with targeted studies, for example, using an inoculum after enrichment or isolated bacteria. Here, degradation of 2‐cyclohexylidene‐2‐phenylacetonitrile (Peonile) was studied. Peonile is composed of biodegradable substructures and does not have typical nonbiodegradable motifs, such as extensive alkyl‐branching or quaternary carbon atoms, yet it fails ready biodegradability tests. An adapted sludge was able to mineralise Peonile with a bi‐phasic curve. Two *Acidovorax* strains isolated from this inoculum degrade the phenyl ring of Peonile, generating the metabolite 2‐cyano‐2‐cyclohexylideneacetic acid. A *Variovorax* strain degrading this metabolite was isolated at the end of a mineralisation experiment with the adapted inoculum. A mixture of both bacteria was shown to mineralise Peonile under OECD 301D and 301F incubation conditions. The need for cooperative action of two bacteria explains the bi‐phasic curve in the mineralisation by the adapted sludge and indicates that Peonile is ultimately biodegradable by bacteria originally present in sewage sludge. This is one of the first examples showing cooperative mineralisation of a xenobiotic by specific bacteria in sewage sludge.

## Graphical abstract

Cooperative mineralisation of the fragrance ingredient Peonile by a consortium of a *Variovorax* and an *Acidovorax* strain isolated from activated sludge from a sewage treatment plant.

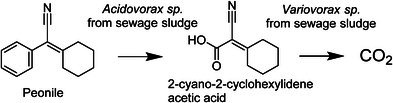

## 1 Introduction

Ultimate biodegradation signifies the ability of bacteria to mineralise organic chemicals by converting the carbon skeleton into CO_2_, and it is a more relevant environmental criterion as compared to primary biodegradation, which describes the loss of a parent compound by at least one metabolic transformation step to form a potentially stable metabolite. To be considered ultimately biodegradable, a substance typically needs to reach the pass level of ≥ 60% oxygen consumption based on the theoretical oxygen demand or carbon dioxide production within a specified timeframe according to international test guidelines such as OECD 301 (OECD 1992). These tests are stringent, and a negative result does not necessarily mean a chemical will not degrade in the environment. Thus, whether a substance is ultimately biodegradable can be further studied with more targeted studies, for example, over a more prolonged time window, using an inoculum after an enrichment culture (Kahl et al. 2018; Zhang et al. 2020) or using specific isolated bacteria (Kleinsteuber et al. 2019; Ma et al. 2020). Such tests do not show biodegradability according to OECD criteria, but they do indicate whether the initial inoculum contains bacteria able to express all relevant enzymes to ultimately degrade a particular substrate. Such data may add to a weight‐of‐evidence assessment on persistency.

The biological inoculum used in OECD tests mainly comes from activated sludge from sewage treatment plants, which contains a high number and diversity of metabolising bacteria. However, typical OECD‐approved screening tests use a high concentration of a substrate in the presence of a diluted inoculum because respiration due to mineralisation of the test substance needs to be clearly higher than the background respiration of the inoculum. This experimental set‐up facilitates degradation of test substances whereby a single bacterial strain can grow on the substrate as the sole carbon source and fully metabolise it. Thus, at the end of OECD 301F tests conducted on biodegradable fragrance compounds and natural terpenes, we typically isolate single bacteria from the biomass which can fully mineralise the test substrate (our own unpublished observation). This is aligned with the fact that the oxygen uptake curves in 301F tests usually are sigmoidal as expected for typical growth curves of single bacteria metabolising a given carbon source. However, evidence from the degradation of some herbicides such as diuron, atrazine, and acetochlor indicates that biodegradation in some cases is due to cooperative mineralisation by two or more bacterial strains, whereby the first strain forms a metabolite further mineralised by a second strain (Smith et al. 2005; Devers‐Lamrani et al. 2014; Hou et al. 2014; Douglass et al. 2017; Li et al. 2021). For example, diuron degrading strains were isolated from contaminated soil and sediment. The collective action of a strain that degrades diuron to 3,4‐dichloroaniline, followed by the action of a strain capable of degrading this metabolite, led to the complete mineralisation of diuron (Devers‐Lamrani et al. 2014). Cooperative metabolisation by a combination of three bacteria was shown for acetochlor (Hou et al. 2014).

The fragrance compound Peonile (2‐cyclohexylidene‐2‐phenylacetonitrile) is composed of chemical substructures that are typically biodegradable (mainly a phenyl and a cyclohexane ring (ECHA 2024, 2025a, 2025b)), and it does not contain typical motifs rendering chemicals nonbiodegradable, such as extensive alkyl‐branching or quaternary carbon atoms; yet it is not biodegradable in a 28‐d OECD 301F screening test (Api et al. 2022). In this study, we investigated whether Peonile is ultimately biodegradable by the bacteria originally present in the inoculum from sewage treatment plants, as used in OECD 301F tests. This was investigated by conducting an enrichment step with prolonged substrate exposure, followed by the isolation and characterisation of Peonile‐degrading strains. A consortium of two strains isolated from the sludge was able to metabolise Peonile through cooperative mineralisation.

## 2 Materials and Methods

### 2.1 Chemicals

Peonile (2‐cyclohexylidene‐2‐phenylacetonitrile) was obtained from Givaudan Switzerland SA. The metabolite 2‐cyano‐2‐cyclohexylideneacetic acid (2CCHA) was prepared in 56% isolated yield by condensation of cyanoacetic acid with cyclohexanone in refluxing toluene (6 h) in the presence of acetic acid (0.2 equiv.) and beta alanine (0.1 equiv.) and recrystallised from toluene. The structure was confirmed by analytical data: ^1^H NMR: 11.51 (br s, 1 H), 2.98–3.03 (m, 2 H), 2.69–2.74 (m, 2 H), 1.65–1.87 (m, 6 H).^13^C NMR: 183.9 (s), 167.4 (s), 115.0 (s), 101.3 (s), 37.5 (t), 32.0 (t), 28.8 (t), 28.5 (t), 25.5 (t). LC–MS (ESI, negative mode): 164.4 [M‐H]^−^.

### 2.2 Collection of Sewage Sludge and Enrichment for Peonile‐Degrading Strains

Fresh activated sludge from a biological wastewater treatment plant treating predominantly domestic sewage (Bois‐de‐Bay, Satigny, Switzerland) was washed three times in OECD mineral medium (MM) (OECD 1992) and kept at 4°C under aerobic conditions until the start of the enrichment. 100 μL of a 75 mg/mL stock solution of Peonile in diethyl ether was added to a 1 L glass flask and allowed to evaporate for 10 min. Then, 5 mL of suspended sludge diluted to a concentration of 1.53 g/L dry matter and 250 mL of MM amended with 1 mL/L trace elements solution (Trace Metal Mix A5 with Co; SIGMA‐Aldrich) were added. The flask was incubated in the dark under constant stirring at room temperature for 28 days. The culture was centrifuged, re‐suspended in fresh MM, and added to a flask with fresh substrate (30 mg/L) and further incubated. A total of three 28‐day enrichment cycles were performed. A parallel control flask was amended with sludge without added substrate. The enrichment was conducted twice on independent sludge samples.

### 2.3 Mineralisation Experiment With Enriched Culture Under OECD 301D Incubation Conditions

The adapted sludge was centrifuged and re‐suspended in fresh MM. An aliquot of the culture was passed through a syringe needle to dissociate bacterial aggregates by shear force. A Peonile stock solution (285 μL) at 5.9 mg/mL in tert‐butyl‐methyl ether was added to test containers (glass flasks with a total volume of 570 mL holding a PreSens oxygen sensor spot; PSt3; PreSens—Precision Sensing GmbH, Regensburg, Germany). The solution was allowed to evaporate for 20 min, and then 559 mL of MM saturated with oxygen and 11.4 mL of adapted sludge inoculum were added to completely fill the flasks to give a final concentration of Peonile of 2.95 mg/L, that is, the concentration that would lead to complete oxygen depletion upon full mineralisation. The flasks were then incubated at 22.5°C in a temperature‐controlled cabinet, and the oxygen saturation was measured constantly with the PreSens Oxygen Measurement System OXY‐4 SMA and the PreSens Measurement Studio 2 Software. Parallel flasks were prepared, having only MM with adapted sludge inoculum and no substrate.

### 2.4 Isolation of Bacteria From Enrichment Culture

The dissociated adapted sludge culture was spread‐plated on 1/10‐strength tryptic soy broth. Isolated colonies after 7 days were re‐streaked several times to obtain pure cultures. For taxonomic assignment, the 16S rRNA gene was amplified with the primers 27F and 1492R [1] and sequenced by Sanger sequencing (Microsynth, Balgach, Switzerland).

### 2.5 Primary Degradation Experiments With Isolated Bacteria

The strains were grown at 22.5°C in liquid tryptic soy broth for 3–5 days, adjusted to an optical density OD_600_ = 1 in MM (1 mL) and incubated with substrates at 30 mg/L at 22.5°C for 1 h–14 days. Primary degradation by individual isolates was measured by extracting the culture with 0.1 mL 1 M HCl and 1.0 mL of methyl‐tert‐butylether (MTBE) containing 5 ppm of dodecane as an internal standard. Analysis was performed in the splitless injection mode on an Agilent 6890N Gas Chromatograph (GC) equipped with a flame‐ionisation detector and an Rtx‐5 w/Integra‐Guard (10224‐125) column (30 m length × 0.32 mm internal diameter × 0.25 μm film thickness). Hydrogen (2 mL/min) was used as carrier gas, starting at a temperature of 60°C held for 1 min and a subsequent linear gradient increase of 10°C per minute up to a temperature of 240°C, which was then held for 10 min. All concentrations were calculated by normalisation to the internal standard dodecane. The % degradation was assessed versus substrate samples without added bacteria, incubated under identical conditions, and extracted at the start and at the end of the experiment. With this method, using acidic extraction, both parent Peonile and acid metabolites can be detected and quantified.

### 2.6 Mineralisation Experiments With Isolated Bacteria Under OECD 301D Conditions

Test bacteria were grown and re‐suspended in MM as described above. Glass vials (21 mL total volume) holding a PreSens oxygen sensor (OxoDish) on the bottom were spiked with a Peonile solution (21 μL, 3 mg/mL in diethyl ether), which was allowed to evaporate. Then, 10 mL MM and 105 μL of a diluted bacterial culture were added (52.5 μL of each strain in cases where a combination of two strains was used), targeting a final bacterial count of 10^6^ cfu/mL. The vials were filled with no air left on the top, capped, and placed on a PreSens SDR (sensor dish reader unit) for continuous online oxygen determination in the test vials with the SDR_V4.0.0 software. To measure the concentration of substrate and to assess how close the measured concentration is to the nominal concentration in this miniaturised biodegradation set‐up, identical parallel glass vials without an oxygen sensor spot were coated with the substrate and filled with MM. The test solution was extracted at *T* = 1 h and analysed by GC as described above. In parallel, 21 μL of the substrate solution was directly spiked into vials with buffer and extracted at once. By comparing the concentration in the coated vials with the directly spiked buffer samples, the loss of parent chemical during the set up of the experiment due to volatility could be calculated, and the actual dosed concentration determined analytically. Since the biodegradation tests are then run under closed conditions, volatility does not further affect the results once the experiments are set up and the flasks are closed.

### 2.7 Mineralisation Experiments With Isolated Bacteria Under OECD 301F Conditions

The respirometer used was an OxiTop control system, made by Wissenschaftlich‐Technische Werkstätten (WTW), Weilheim, Germany. Bacteria were cultivated as described above and re‐suspended in MM. Test substance samples (7.6 mg of the neat chemical, corresponding to 30 mg/L in 255 mL of test medium) were weighed in small aluminium boats and added directly to the test flasks, which were filled with 2.0 mL of the bacterial suspension (each 1 mL in case two strains are combined) and 250 mL of MM (final bacterial density of 2 × 10^7^ cfu/mL). By dosing the neat chemical without a solvent and evaporation step, substance loss due to evaporation can be avoided in this set up. Two sodium hydroxide pellets were placed in the quivers on top of the bottles, which were closed tightly with the OxiTop measuring heads and allowed to equilibrate to the test temperature (22°C ± 1°C) in the temperature‐controlled OxiTop system before the start of stirring and daily data acquisition of oxygen consumption and temperature. Oxygen uptakes, as read on the OxiTop controller, were based on the exact weighted amount of added test substances.

### 2.8 Detection of Metabolites

The test substances were dissolved in DMSO at 6 mg/mL, and 5 μL of the solution was added to 10 mL glass vials with 1 mL of an OD_600_ culture. After different time intervals, samples were heated to 80°C for 1 min and frozen at −80°C to stop the reaction. Samples were sonicated in an ice bath for 10 s with a Branson sonicator equipped with a micro‐tip, centrifuged at 4000 rpm at 4°C for 10 min, and the supernatant loaded onto solid phase extraction cartridges (OASIS HLB μElution plate; conditioned and equilibrated with 200 μL of methanol and 200 μL of H_2_O). Columns were washed with 200 μL of 5% methanol in H_2_O, and samples eluted sequentially with 50 μL methanol and 50 μL H_2_O and analysed by high resolution LC–MS (LC‐HRMS) on a Dionex UltiMate 3000 RS HPLC system coupled to a Q Exactive Orbitrap mass spectrometer (Thermo Scientific, Reinach, Switzerland) with electrospray ionisation (ESI) in positive and negative ionisation modes. For liquid chromatography separation, an Acquity UPLC Peptide BEH C18 column with a pore size of 130 Å, dimensions 2.1 × 100 mm, and particle size of 1.7 μm was used with a 2.1 × 5 mm pre‐column with a particle size of 1.7 μm of the same material. The flow rate was 0.25 mL/min. Eluent A consisted of water containing 5% methanol and 0.1% formic acid, and eluent B consisted of methanol containing 5% water and 0.1% formic acid. A linear gradient was run from 100% eluent A (hold for 0.5 min) to 100% eluent B within 5 min (hold for 3 min), back to 100% eluent A within 0 min followed by 1.5 min equilibration time. The injection volume of the sample was 10 μL. The mass resolution of the HR‐MS spectra was set to 70,000. The mass accuracy was < 5 ppm. Data‐dependent high resolution product ion spectra (HR‐MS/MS) were recorded at a resolution of 17,500. Ion source parameters adjusted were as follows: sheath gas flow (46 arbitrary unit), auxiliary gas flow (11 arbitrary unit), sweep gas flow (2 arbitrary unit), capillary temperature (253°C), and source voltage (3.5 kV in positive mode and 2.5 kV in negative mode). Fragmentation was obtained from dissociation in an octopole collision cell using higher energy collision dissociation settings at NCE = 35 (arbitrary unit). The mass scan range was set from 50 to 750 m/z.

## 3 Results

### 3.1 Enrichment Culture and Mineralisation Experiment With Adapted Sludge Under OECD 301D Conditions

**FIGURE 1:**
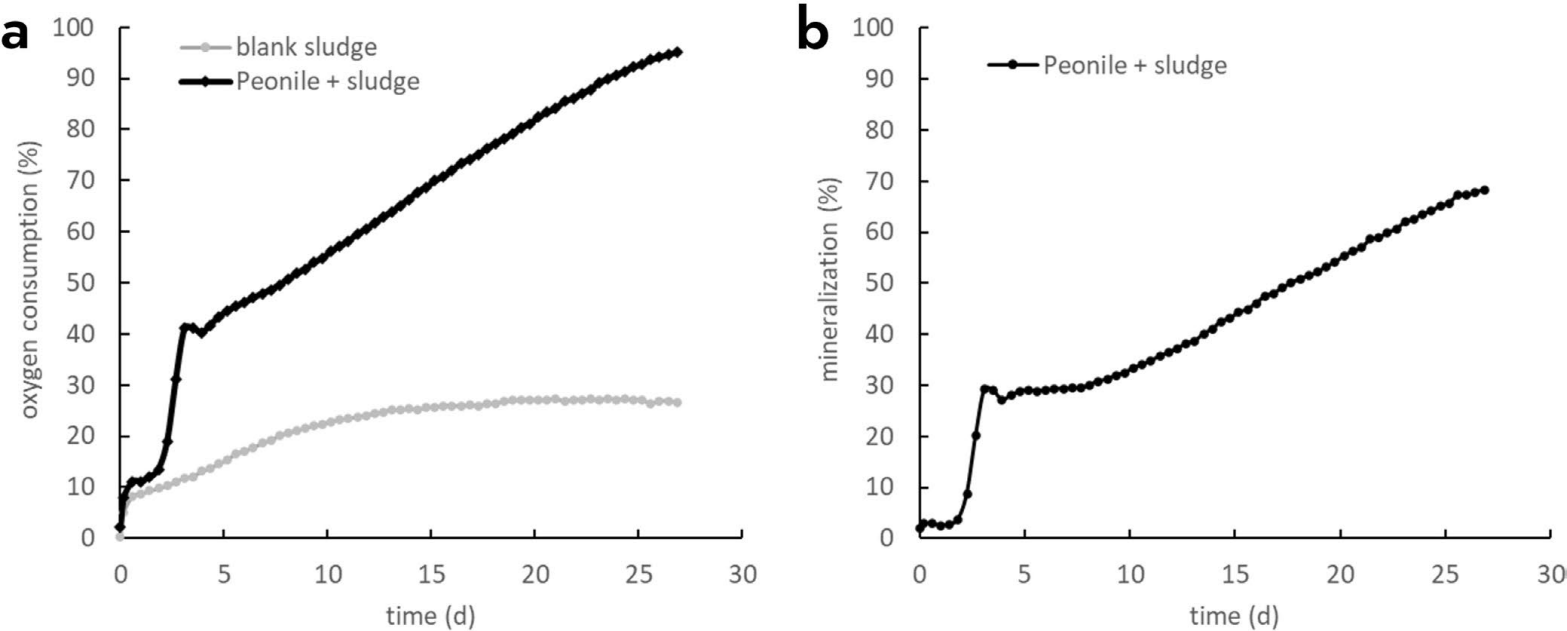
Degradation of Peonile by the original adapted sludge culture. (a) Oxygen consumption from oxygen‐saturated buffer by sludge adapted to Peonile for three 28‐day cycles in the absence (grey symbols) and the presence (black symbols) of Peonile. (b) Respiration by blank adapted sludge was subtracted to calculate the % mineralisation of Peonile. Shown are the data for the original sludge from which the bacterial strains were isolated.

Continuous exposure of sludge to Peonile over three 28‐day cycles led to visible biomass accumulation and increased bacterial counts. The bacterial counts in control sludge without substrate addition but incubated under identical conditions after the enrichment were at 1 × 10^5^ cfu/mL, while in the Peonile adapted culture the counts reached 1.3 × 10^7^ cfu/mL. The resulting adapted inoculum was diluted 50‐fold in MM and assessed for mineralisation of Peonile at a concentration of 2.95 mg/L. As shown in Figure 1, the diluted enrichment culture showed a rapid uptake of oxygen in the presence of Peonile over the first three days, then biodegradation plateaued for a few days before resuming at a clearly slower rate. After subtraction of the respiration by blank adapted sludge, 70% mineralisation was reached after 27 days. The bi‐phasic curve pointed to two separate processes in this degradation process, with rapid initial degradation followed by a slower degradation rate.

### 3.2 Isolation and Characterisation of Bacterial Strains Degrading Peonile

**FIGURE 2:**
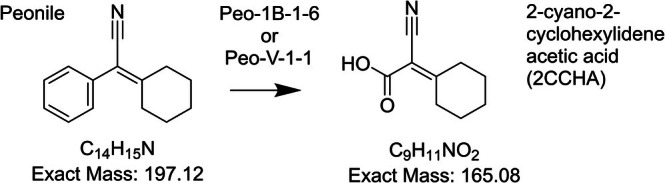
Proposed structure of metabolite with MW of 165.08 Da found in incubations of Peonile with the bacterial isolates Peo‐1B‐1‐6 and Peo‐V‐1‐1.

**FIGURE 3:**
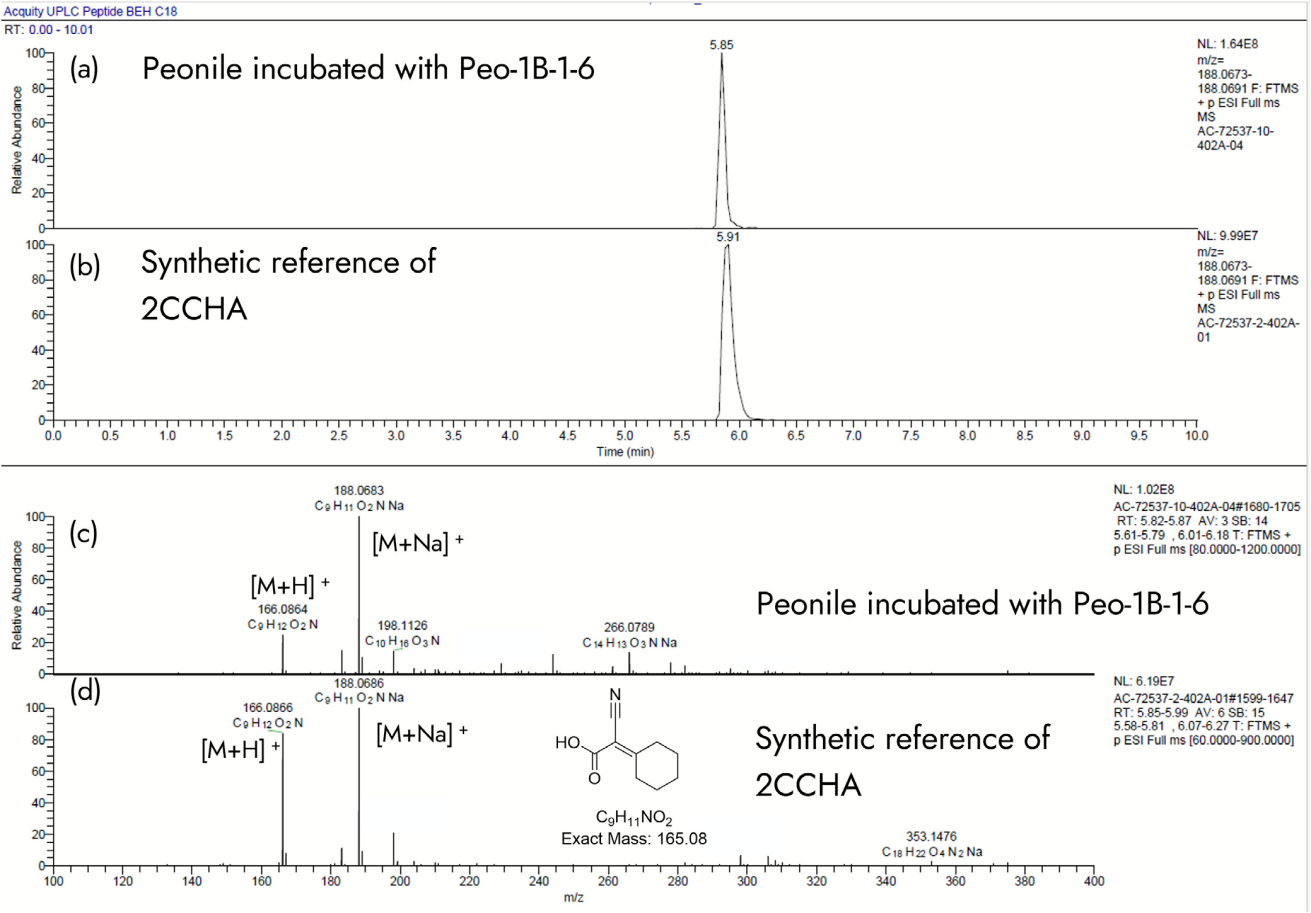
Formation of a stable metabolite by Peo‐1B‐1‐6. Extract from Peo‐1B‐1‐6 incubated with Peonile after 14 days (a and c) and synthetic 2CCHA (b and d) as analysed by HR LC–MS analysis. Extracted chromatogram for the [M + Na]^+^ ion with 188.06 Da (a and b) and MS of the peak at RT 5.9 (c and d).

Single colonies from the adapted sludge plated on 1/10 tryptic soy agar were picked, purified, and further assessed for their taxonomic assignment. Two isolated strains (Peo‐1B‐1‐6 and Peo‐V‐1‐1) from two independent enrichment cultures were able to perform primary degradation of Peonile and were therefore studied further. Both belong to the group of *Acidovorax* sp. based on 16S RNA sequence and are closely related to each other. These strains perform 99% primary degradation of Peonile within 14 days while accumulating a stable metabolite, which was detected by GC after extraction of the acidified samples. Based on high‐resolution LC–MS analysis, it was shown to have a molecular weight of 165.08 Da (Figure 3). It was hypothesised that these strains degrade the benzyl ring, leaving a carboxylic acid, as shown in Figure 2. The putative metabolite 2‐cyano‐2‐cyclohexylideneacetic acid (2CCHA) was therefore synthesised and analysed in parallel to the extract from the incubation of Peonile with strain Peo‐1B‐1‐6, as shown in Figure 3. LC–MS analysis confirmed that the synthetic sample and the extracted metabolite have identical MS‐spectra and the same retention time.

Mineralisation of Peonile by strain Peo‐1B‐1‐6 was further evaluated under dilute conditions of an OECD 301D test with measurement of oxygen uptake in oxygen‐saturated MM. Strain Peo‐1B‐1‐6 resulted in a maximum of 30% oxygen consumption, which agrees with its ability to mineralise Peonile only partially, leading to the formation of the metabolite 2CCHA and mineralisation of the five carbon atoms of the benzyl ring (data not shown). Because (i) the bacterial strains so far isolated were only able to partially mineralise Peonile and led to the formation of a metabolite and (ii) the original adapted sludge experiment showed a bi‐phasic behaviour (Figure 1), it was hypothesised that a second bacterial strain must be able to complete the second part of the mineralisation pathway, that is, the second and slower phase of oxygen uptake. Therefore, the experiment shown in Figure 1 was repeated with a defrosted aliquot of the adapted sludge and, at the end of the mineralisation experiment, further bacterial isolates were collected. We expected that at the end of the second phase of the mineralisation, bacteria specialised for the terminal mineralisation steps would be more abundant. From these diluted cultures after mineralisation of Peonile by the adapted sludge, a third bacterial strain was isolated (Peo‐22‐4). By 16S rDNA analysis, it was identified as a member of *Variovorax* sp. (most closely related to *Variovorax boronicumulans*).

### 3.3 Primary Degradation Experiments With Isolated Bacteria and Combinations of Bacteria

The different strains isolated from adapted sludge (Peo‐1B‐1‐6 and Peo‐V‐1‐1) or from mineralisation experiments with adapted sludge (Peo‐22‐4) were incubated with Peonile or the stable metabolite 2CCHA. Single strains and combinations of strains were evaluated. As shown for two independent experiments in Tables S1 and S2, Peo‐1B‐1‐6 and Peo‐V‐1‐1 both lead to complete primary degradation of Peonile and the accumulation of 2CCHA within 14 days. On the other hand, the strain Peo‐22‐4 leads to complete primary degradation of 2CCHA, but not of Peonile. Combining the strain Peo‐22‐4 with either Peo‐1B‐1‐6 or Peo‐V‐1‐1 leads to degradation of Peonile without accumulation of the intermediate metabolite 2CCHA, showing that this combination of strains can further degrade the metabolite.

Data in Tables S1 and S2 is from experiments conducted over 14 d. In a more detailed experiment, Peo‐1B‐1‐6 was assessed alone and in combination with Peo‐22‐4 for 1 and 4 h only (Table 1). Complete primary degradation of Peonile by Peo‐1B‐1‐6 and of 2CCHA by Peo‐22‐4 was seen within 1 h, showing that this process is fast in static cultures at OD_600_ = 1. The combination of Peo‐1B‐1‐6 and Peo‐22‐4 led to complete primary degradation of Peonile within 1 h with partial accumulation of the metabolite, while after 4 h both parent and metabolite were completely metabolised. Peo‐22‐4 completely metabolised 2CCHA within 1 h (Table 1).

**TABLE 1:** Primary degradation of Peonile and its metabolite 2CCHA by the isolated strains after short incubation time.

Strain 1	Strain 2	Time (h)	Substrate	Peonile remaining (%)^a^	2CCHA remaining (%)^a^, ^b^
*Blank*	—	1	Peonile	92 ± 3.8^c^	0 ± 0
*Blank*	—	4	Peonile	95 ± 2.3	0 ± 0
Peo‐1B‐1‐6	—	1	Peonile	1 ± 0.4	97.9 ± 3.1
Peo‐1B‐1‐6	—	4	Peonile	0.8 ± 0	111.7 ± 7.7
Peo‐1B‐1‐6	Peo‐22‐4	1	Peonile	6.3 ± 2	56.4 ± 6.1
Peo‐1B‐1‐6	Peo‐22‐4	4	Peonile	0.7 ± 0	0 ± 0
*Blank*	—	1	2CCHA	—	113.4 ± 4.7
*Blank*	—	4	2CCHA	—	103.3 ± 2.7
Peo‐22‐4	—	1	2CCHA	—	0 ± 0
Peo‐22‐4	—	4	2CCHA	—	0 ± 0

^a^ % of the amount of Peonile or 2CCHA dosed at 30 mg/L at the start of the experiment and as detected by GC‐FID analysis.

^b^ in the case of 2CCHA as a metabolite of Peonile, % relates to the equimolar amount theoretically formed from Peonile.

^c^ Shown are averages and standard deviations of an experiment conducted in triplicates. Similar experiments conducted over 14 days are shown in Tables S1 and S2.

### 3.4 Mineralisation Experiments With Isolated Bacteria Under OECD 301D Conditions

**FIGURE 4:**
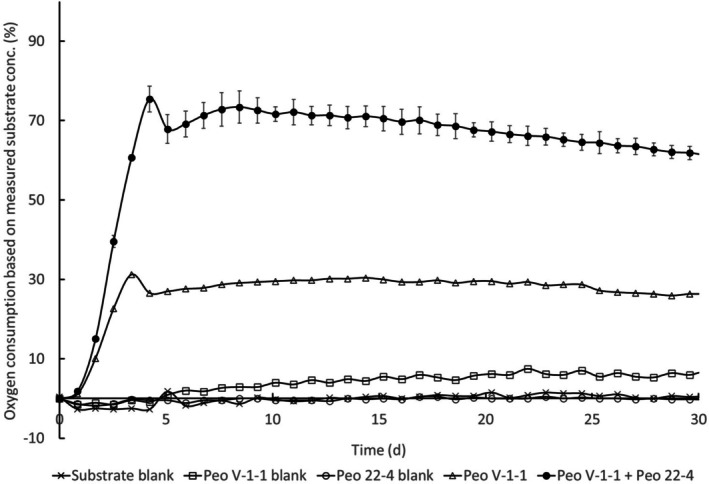
Oxygen consumption (% of theoretical oxygen demand) of dilute cultures of the strain Peo‐V‐1 and Peo‐22‐4 and their combination without substrate (blank, open squares and open circles) and in the presence of Peonile (open triangles and closed circles). Data are presented when calculated based on measured substrate concentration; Figure S1 reports the uncorrected values. Data for the consortium of Peo‐V‐1‐1 and Peo 22–4 are from duplicates within the experiment, while the other treatments were run once. An independent repetition with the combination of Peo 22–4 with Peo 1B‐1‐6 is shown in Figure S2.

The strains Peo‐V‐1‐1 and Peo‐1B‐1‐6, and combinations with strain Peo‐22‐4, were further evaluated for mineralisation of Peonile in the miniaturised 301D system measuring consumption of dissolved oxygen. The nominal substrate concentration was adjusted such that 100% mineralisation would lead to 100% oxygen consumption in these 21 mL test vials. However, due to the volatility of the substrate, it was found that the actual substrate concentration after coating the vials in the miniaturised set‐up with the substrate solution and after evaporation of the solvent was lower than the nominal concentration (ca. 68% of the nominal concentration), and hence data are expressed both based on measured (Figure 4) and nominal concentration (Figure S1). The bacterial strain Peo‐V‐1 gave only around 30% mineralisation. The combination with Peo‐22‐4 led to full mineralisation (> 60% if expressed based on measured substrate concentration). A similar result was obtained with the combination of Peo‐22‐4 with Peo‐1B‐1‐6 (Figure S2). Strain Peo‐22‐4 was also evaluated on its own for mineralisation of 2CCHA in the miniaturised 301D system, measuring consumption of dissolved oxygen. It reached 50% mineralisation (based on measured substrate concentration), as shown in Supporting Information, Figure S3.

### 3.5 Mineralisation Experiments With Isolated Bacteria Under OECD 301F Incubation Conditions

Since actual and nominal concentrations of Peonile differed under the 301D conditions in the miniaturised system, further tests were conducted at higher substrate loading under standard OECD 301F conditions and with oxygen consumption measured with standard OxiTop heads. In this experimental set‐up, the substrate is directly weighed and added without a solvent and an evaporation step. Two independent experiments were performed. In the first, both strains Peo‐1B‐1‐6 and Peo‐V‐1 were evaluated individually and in combination with Peo‐22‐4, while in the second, only strain Peo‐1B‐1‐6 was re‐tested and combined with Peo‐22‐4.

**FIGURE 5:**
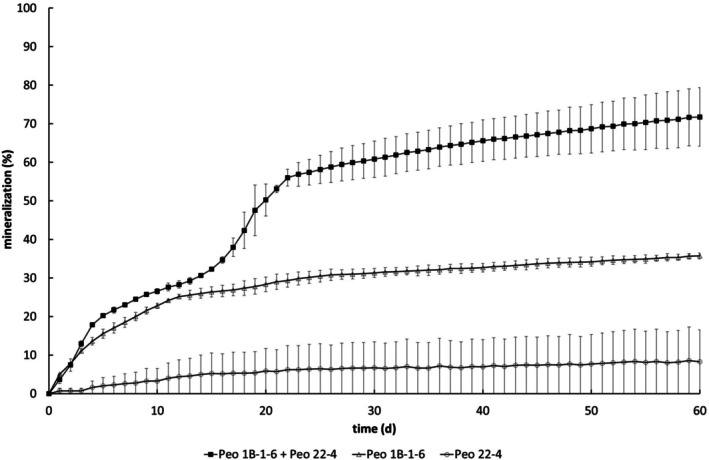
Biodegradation of Peonile by the bacterial isolates Peo‐1B‐1‐6 and a combination of Peo‐1B‐1‐6 and Peo‐22‐4. Measured with OxiTop heads under OECD 301F conditions (30 mg/L substrate). % Mineralisation refers to % of theoretical oxygen demand consumed after subtraction of the oxygen consumption by cultures without substrate. Data from a representative experiment with duplicate samples is shown. An independent replication is shown in Figure S4; an independent replication with the combination of Peo‐V‐1‐1 and Peo‐22‐4 is shown in Figure S5.

As shown in Figure 5, Peo‐1B‐1‐6 led to a rapid start of the biodegradation, but then the degradation plateaued at around 36% biodegradation until the end of the experiment (60 days, Experiment 1). A similar plateau of 28% was reached in the second experiment (Supporting Information, Figure S4).

Addition of strain Peo‐22‐4 led to complete mineralisation as shown by 60% relative oxygen demand after 28 days, and 72% after 60 days in the first experiment and 60% biodegradation after 28 days, and 64% after 40 days in the second experiment. As shown in Figure S5, a similar result was obtained by combining strain Peo‐V‐1‐1 with Peo‐22‐4. Peo‐V‐1‐1 led to a rapid degradation up to a stable plateau (22% at 8 days and 29% at 43 days), while the addition of Peo‐22‐4 led to 60% biodegradation after 28 days and 72% after 43 days.

### 3.6 Analysis for Transient Metabolites

**FIGURE 6:**
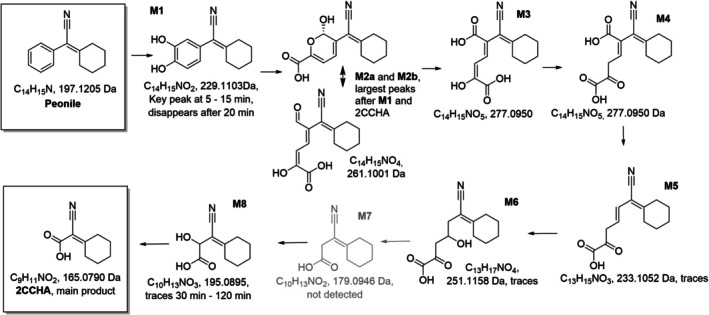
Incubation of Peonile with Peo‐1B‐1‐6 for 5–120 min and analysis for potential transient metabolites by HR LC–MS. These metabolite masses were only seen in incubations with Peo‐1B‐1‐6 and the substrate, and not in the controls with bacteria only. The structures of M1—M8 are tentative and based only on the exact mass as observed by HR LC–MS and thus require further structural confirmation. The mass of putative M7 in grey was not observed.

To further understand the potential biodegradation pathway, an experiment with Peo‐1B‐1‐6 at OD_600_ = 1 in MM was conducted, and samples were taken at short time intervals (5, 10, 15, 20, 30, 60, 120 min, 240 min, 1 and 7 days) after substrate addition and analysed with HR LC–MS. The same experiment was also done with 2CCHA incubated with Peo‐22‐4. When Peonile was incubated with strain Peo‐1B‐1‐6, very rapidly (5 min after the start of the experiment) a metabolite peak appeared with an exact mass of 229.11 and a theoretical molecular formula of C_14_H_15_NO_2_, most probably indicating the formation of the catechol at the phenyl ring (M1, Figure 6). The second most abundant metabolite, with a molecular formula of C_14_H_15_NO_4,_ is present as two peaks and is consistent with the structures M2a and M2b, that is, the open and the hemiacetal form. This metabolite was most abundant after 20 min. In addition to these two most abundant transient metabolites, further metabolites with theoretical molecular formulas of C_14_H_17_NO_5_, C_13_H_15_NO_3_, C_13_H_17_NO_4_, and C_10_H_13_NO_3_ were observed. These metabolites occurred at low levels (< 1% of total peak area), but only in the presence of substrate and bacteria, and not in the controls with bacteria only. An exact structure assignment was not possible based on LC–MS only, and the low transient quantity does not allow for isolation and NMR analysis; hence, the molecular structures of M1—M8 in Figure 6 are tentative. However, this molecular formula would be congruent with the degradation of the phenyl ring by the *meta*‐cleavage pathway outlined in Figure 6. This degradation occurs very rapidly, and the metabolites do not accumulate. Already after 30 min, the Peonile substrate is almost quantitatively transformed into the main metabolite, namely, 2CCHA, as confirmed by LC–MS and in agreement with the experiment in Table 1.

When 2CCHA was incubated with Peo‐22‐4 and analysed by LC–MS, the substrate was quantitatively metabolised within 60 min (Supporting Information, Figure S6; confirming the results in Table 1), however, no major metabolites accumulated as detected by LC–MS, neither in positive nor in negative ionisation mode (see Figure S6). As an exception, a very low amount (less than 2% total peak area) of a metabolite with a theoretical molecular formula of C_8_H_12_O_3_ was detected, which would correspond to cleavage of the nitrile by a nitrilase followed by decarboxylation and then hydroxylation at any position (data not shown). Overall, these data show that when the first metabolic step of the second phase of Peonile mineralisation by strain Peo 22‐4 starts, mineralisation is rapid without accumulation of further metabolites, showing that the first step in this degradation is rate‐limiting.

## 4 Discussion

Peonile is not biodegradable in ready biodegradation tests. However, based on biodegradable substructures and the absence of typical motifs making chemicals nonbiodegradable, the possibility of mineralisation by bacteria present in sewage treatment plants appeared plausible. Indeed, an inoculum adapted for a prolonged time can degrade Peonile with a clear bi‐phasic curve. However, bacteria initially isolated from this adapted sludge were only able to degrade the phenyl ring, thereby generating the metabolite 2CCHA. Only by screening bacteria isolated at the end of a mineralisation experiment conducted with this adapted sludge was a bacterial strain able to degrade the metabolite 2CCHA found. Consistent with the bi‐phasic curve observed initially, a combination of two strains can completely mineralise Peonile, performing the first and the second part of the pathway with different kinetics. This was shown both by the lack of metabolite formation in incubations with a mixture of the two strains in rested cell suspensions, but also by mineralisation experiments under dilute 301D‐like conditions and standard OECD 301F tests measuring consumption of oxygen. A detailed time‐resolved analysis of the metabolism by these two strains confirmed that the Peonile‐degrading strain (e.g., Peo‐1B‐1‐6) very rapidly forms the catechol derivative, and then the metabolism progresses rapidly to form 2CCHA, with only low quantities of transient metabolites detected. Still, the exact mass of these intermediary metabolites suggests that degradation of the initially formed catechol most likely progresses through the *meta*‐cleavage pathway. Similarly, the degradation of 2CCHA by Peo‐22‐4 is rapid with almost complete loss of parent over 60 min incubation and only traces of detectable metabolites seen by LC–MS analysis. Although Peonile is not mineralised under standard OECD 301 test conditions and therefore does not pass the OECD criteria for ready or inherent biodegradability, the evidence presented here shows that strains able to perform the biodegradation pathway are present in sludge from sewage treatment plants, and that these isolates can metabolise the transient metabolite 2CCHA. These data show that Peonile has an intrinsic, ultimate biodegradability as all the metabolic reactions for full mineralisation are present in the sewage sludge. It appears that under the dilute conditions of the OECD tests, the bacteria do not get sufficiently enriched to allow for this mineralisation to occur within the time window of OECD screening tests.

Biodegradation of complex substrates by consortia of bacteria, each performing only a part of the biodegradation sequence, has been studied for a limited number of examples, and in most cases, the bacteria were isolated from a consortium able to degrade the substrate of interest after initial enrichment, like the approach taken in our study. However, for the well‐studied examples of cooperative degradation of herbicides such as diuron (Devers‐Lamrani et al. 2014; Li et al. 2021), atrazine (de Souza et al. 1998; Smith et al. 2005; Douglass et al. 2017), and acetochlor (Hou et al. 2014), the bacterial consortia were enriched from soil contaminated with the respective pesticides; therefore, an in situ enrichment due to prolonged exposure to the pesticides had taken place even prior to the experiments. On the other hand, several studies used enrichment to obtain bacterial consortia from activated sludge with the ability to degrade xenobiotics (Ma et al. 2019; Fernandes et al. 2020; Aguilar‐Romero et al. 2024). However, to our knowledge, the isolation of specific bacterial strains and proof of cooperative metabolisation of a given substrate by multiple strains isolated from sewage sludge, as used in OECD screening tests, has been little studied. In the surprising finding that bacteria in sewage treatment plants had acquired around the year 2010 the ability to mineralise the sweetener acesulfame (Kahl et al. 2018), it was speculated that catabolism is due to cooperative metabolism, as it was initially not possible to isolate pure cultures capable of growing on the substrate. However, further investigating the adapted inoculum, it was found that acesulfame can indeed be mineralised by single bacterial strains isolated from the adapted consortium (Kleinsteuber et al. 2019). Ma et al. also first reported a stable consortium degrading the antibiotic chloramphenicol (Ma et al. 2019), and only later isolated a single strain from this consortium which then was able to completely degrade the substrate (Ma et al. 2020). Thus, in these studies, the cooperative ability of the consortium was finally reduced to single strains. In a study on the degradation of norbornane derivatives, partial degradation with accumulation of a stable metabolite was reported (Seyfried et al. 2015). Follow‐up testing of the metabolite under 301F tests then proved mineralisation of the metabolite, implying that the second step is performed by a different strain or consortium, and this led to the conclusion that the parent compounds are ultimately biodegradable. However, in that case, no bacterial strains were isolated, and the cooperative metabolism by specific bacterial strains was thus not demonstrated (Seyfried et al. 2015).

Given the high density and high diversity of bacterial communities in sewage treatment plants, cooperative metabolism whereby bacteria pick up metabolites formed after initial degradation by other bacteria might be more widespread than the relatively low number of publications on that topic might indicate, and it may not often be observed in the OECD screening tests where a relatively low bacterial inoculum is challenged with a high load of a single substrate. Indeed, the conditions of OECD screening tests may not always be favourable for cases where biodegradation is only possible due to such cooperative action of multiple bacteria.

Further investigating the importance of cooperative metabolism in the degradation of natural and xenobiotic organic chemicals in sewage treatment plants is thus an interesting and challenging task.

## Author Contributions

A.N. devised the study, T.H. conducted all microbiological experimental work and biodegradation studies; A.M. conducted biodegradation studies, Y.W.‐S. conducted LC‐MS analysis, A.N., K.J. and G.K. wrote and revised the manuscript.

## Funding

This work was supported by Givaudan SA.

## Conflicts of Interest

Authors are employees of Givaudan, a company manufacturing Peonile. They received no other incentives for this work but their salary.

## Supporting information


**Data S1:** Supporting Information.

## Data Availability

The data that supports the findings of this study are available in the Supporting Information of this article.
