# Supplementary material for: Cooperative Mineralisation of the Fragrance Ingredient 2‐Cyclohexylidene‐2‐Phenylacetonitrile by a Consortium of a Variovorax and an Acidovorax Strain Isolated From Activated Sludge
Source: Environ Microbiol Rep. 2026 Feb 4;18(1):e70257. doi: 10.1111/1758-2229.70257 (PMC12873448; doi:10.1111/1758-2229.70257)
Supplement: Supplementary file 1 — Data S1: Supporting Information. [file EMI4-18-e70257-s001.docx]

Supporting information to:

Cooperative mineralization of the fragrance ingredient 2-cyclohexylidene-2-phenylacetonitrile by a consortium of a *Variovorax* and an *Acidovorax* strain isolated from activated sludge

Tina Haupt, Arturo Mendoza, Yumiko Weiner-Sekiya*,* Karen Jenner, Georg Kreutzer^2^ and Andreas Natsch

Table S1. Primary degradation of Peonile and its metabolite 2CCHA by bacteria isolated from adapted sludge or from mineralization experiments with adapted sludge.

| **Strain 1** | **Strain 2** | **Time**  **(d)** | **Substrate** | **Peonile remaining (%) ^1)^** | **2CCHA (%) ^1,2)^** |
| --- | --- | --- | --- | --- | --- |
| *Blank* | - | 14 | Peonile | 98.5 ^3)^ |  |
| Peo-1B-1-6 | - | 14 | Peonile | 0.9 | 91.0 |
| Peo-V-1-1 | - | 14 | Peonile | 0.9 | 97.7 |
| Peo-22-4 | - | 14 | Peonile | 97.5 | 0 |
| Peo-1B-1-6 | Peo-22-4 | 14 | Peonile | 0.8 | 0 |
| Peo-V-1-1 | Peo-22-4 | 14 | Peonile | 1.1 | 0 |
| *Blank* | - | 14 | 2CCHA | - | 99.0 |
| Peo-1B-1-6 | - | 14 | 2CCHA | - | 95.6 |
| Peo-V-1-1 | - | 14 | 2CCHA | - | 94.0 |
| - | Peo-22-4 | 14 | 2CCHA | - | 5.1 |

^1)^ % of the amount of Peonile or 2CCHA dosed at 30 mg / L at the start of the experiment and as detected by GC-FID analysis

^2)^ in the case of 2CCHA as a metabolite of Peonile, % relates to the equimolar amount theoretically formed from Peonile

Table S2. Primary degradation of Peonile and its metabolite 2CCHA by bacteria isolated from adapted sludge or from mineralization experiments with adapted sludge. Independent replication of data in Table S1.

| **Strain 1** | **Strain 2** | **Time**  **(d)** | **Substrate** | **Peonile (%) ^1)^** | **2CCHA (%) ^1,2)^** |
| --- | --- | --- | --- | --- | --- |
| *Blank* | - | 14 | Peonile | 89.9 |  |
| Peo-1B-1-6 | - | 14 | Peonile | 1.1 | 98.1 |
| Peo-V-1-1 | - | 14 | Peonile | 1.0 | 39.3 |
| Peo-1B-1-6 | Peo-22-4 | 14 | Peonile | 1.0 | 0.0 |
| Peo-V-1-1 | Peo-22-4 | 14 | Peonile | 1.3 | 0.0 |
| *Blank* | - | 14 | *2CCHA* | - | 96.1 |
| - | Peo-22-4 | 14 | *2CCHA* | - | 0.0 |

^1,2)^ For footnotes, see Table S1


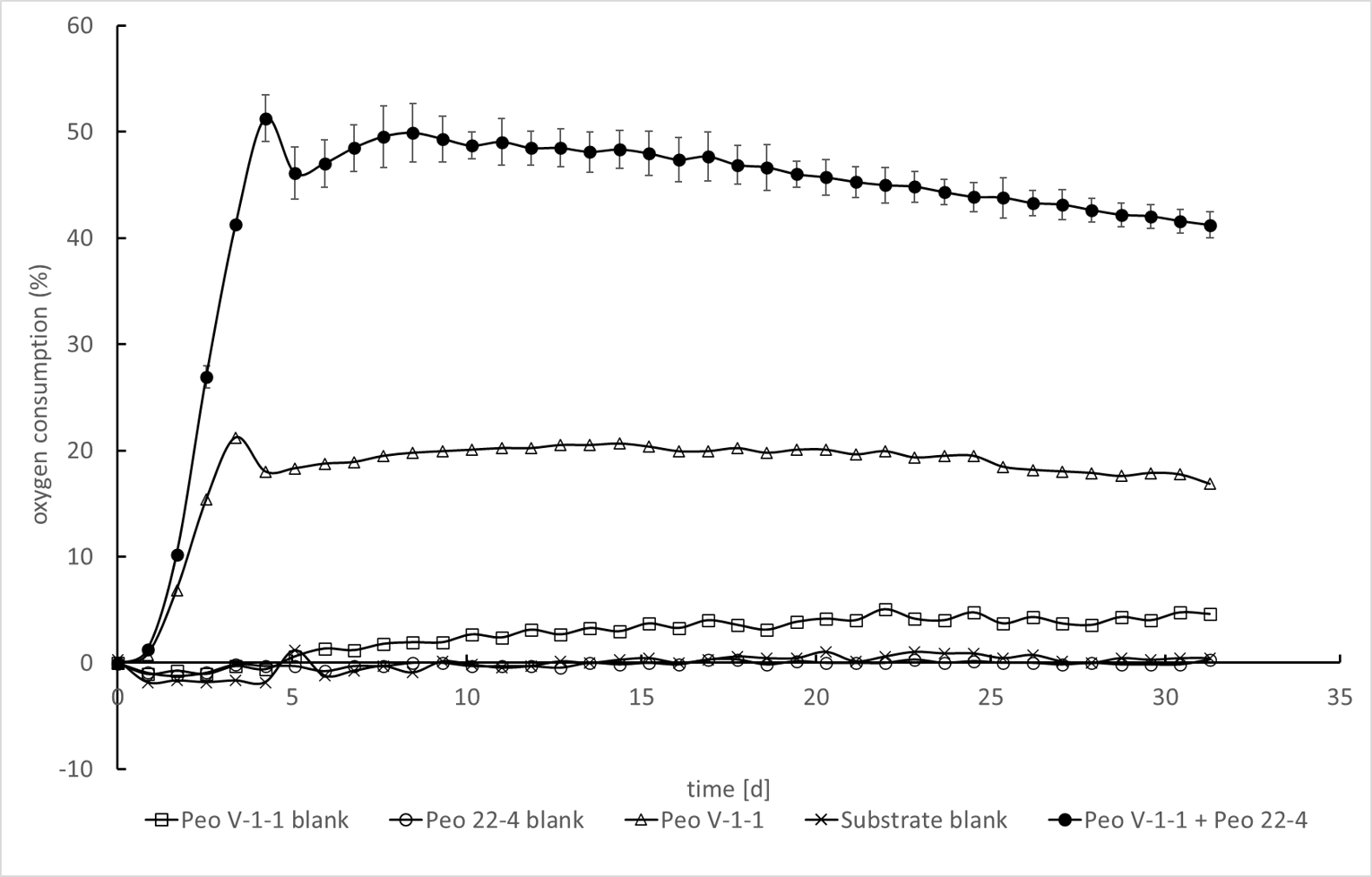


Figure S1. Oxygen consumption (% of theoretical oxygen demand) of dilute cultures of the strain Peo-V-1 and Peo-22-4 without (blank) and in presence of peonile. Data are presented when calculated based on nominal substrate concentration, Figure 4 reports the same data based on actual measured substrate concentration. Singlicate for controls and average and standard deviations from duplicate values for the combination of the two strains.


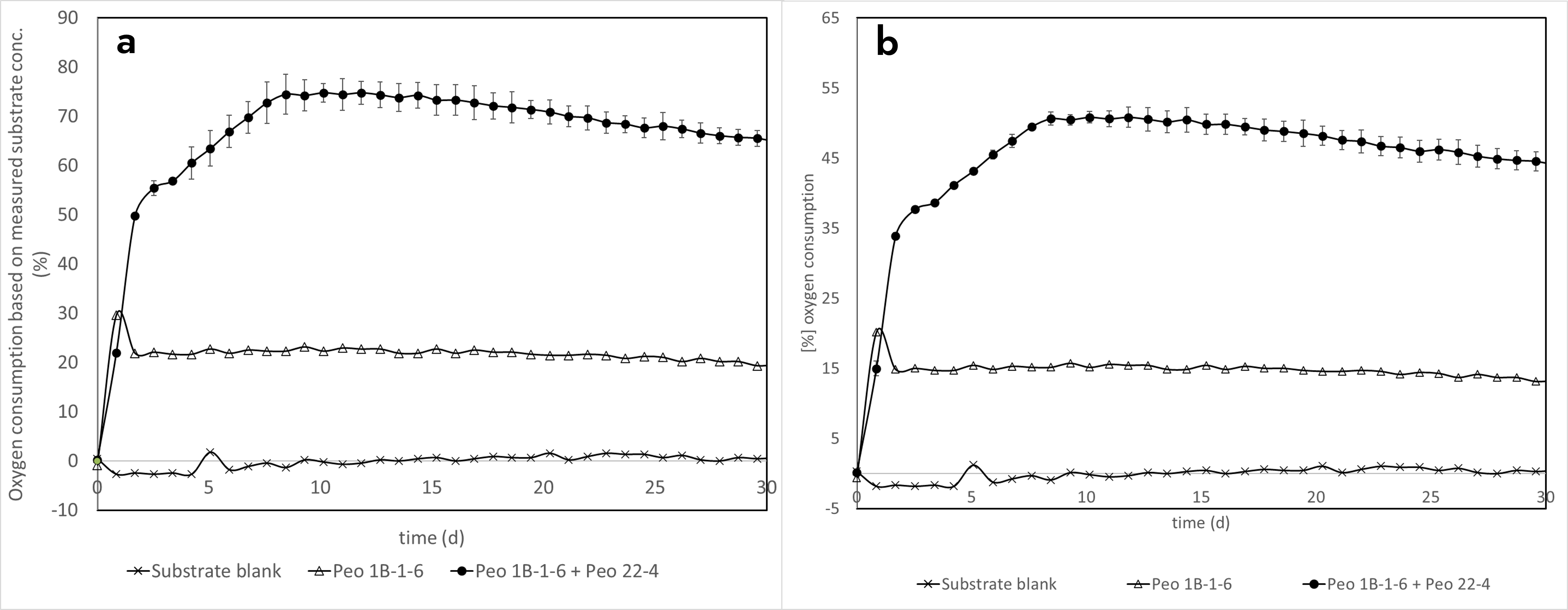


Figure S2. Oxygen consumption (% of theoretical oxygen demand) of dilute cultures of the strain Peo-1B-1-6 and Peo-22-4 in presence of peonile. Data are presented when calculated based on (a) actual measured substrate concentration and (b) on nominal substrate concentration. Singlicate for controls and average and standard deviations from duplicate values for the combination of the two strains.


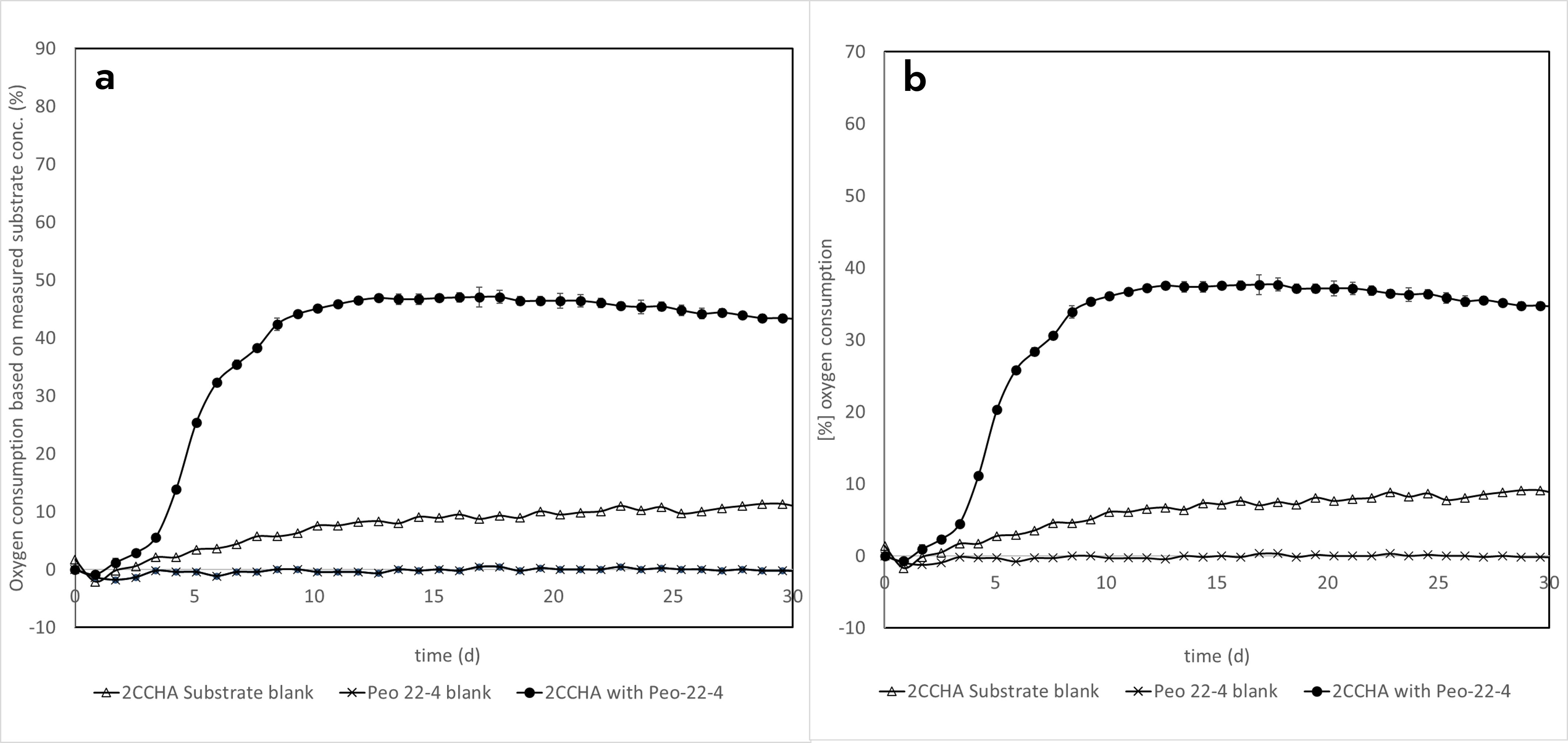


Figure S3. Oxygen consumption (% of theoretical oxygen demand) of dilute cultures of the strain Peo- Peo-22-4 without (blank) and in presence of 2CCHA. Data are presented when calculated based on (a) actual measured substrate concentration and (b) on nominal substrate concentration. Singlicate for controls and average and standard deviations from duplicate values for the combination of the two strains.


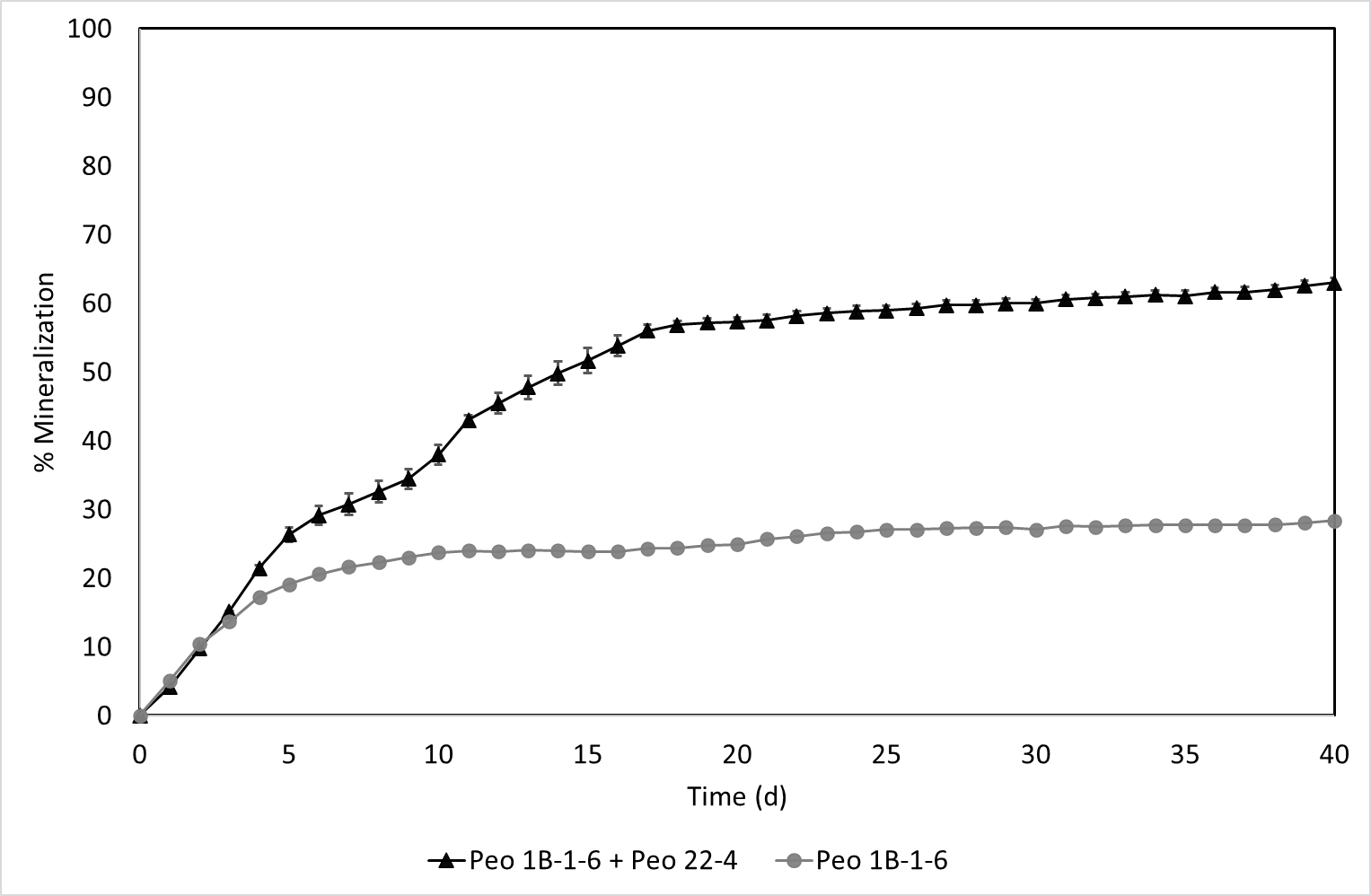


Figure S4. Biodegradation of peonile by the bacterial isolates Peo-1B-1-6 and a combination of Peo-1B-1-6 and Peo-22-4. Independent replication of the experiment in main manuscript Figure 5. Measured with OxiTop heads under OECD 301F conditions (30 mg / L substrate). % Biodegradation refers to % of theoretical oxygen demand consumed after subtraction of the oxygen consumption by bacterial inoculum without substrate. Duplicate cultures for the consortium and singlicate for the strain Peo1B-1-6.


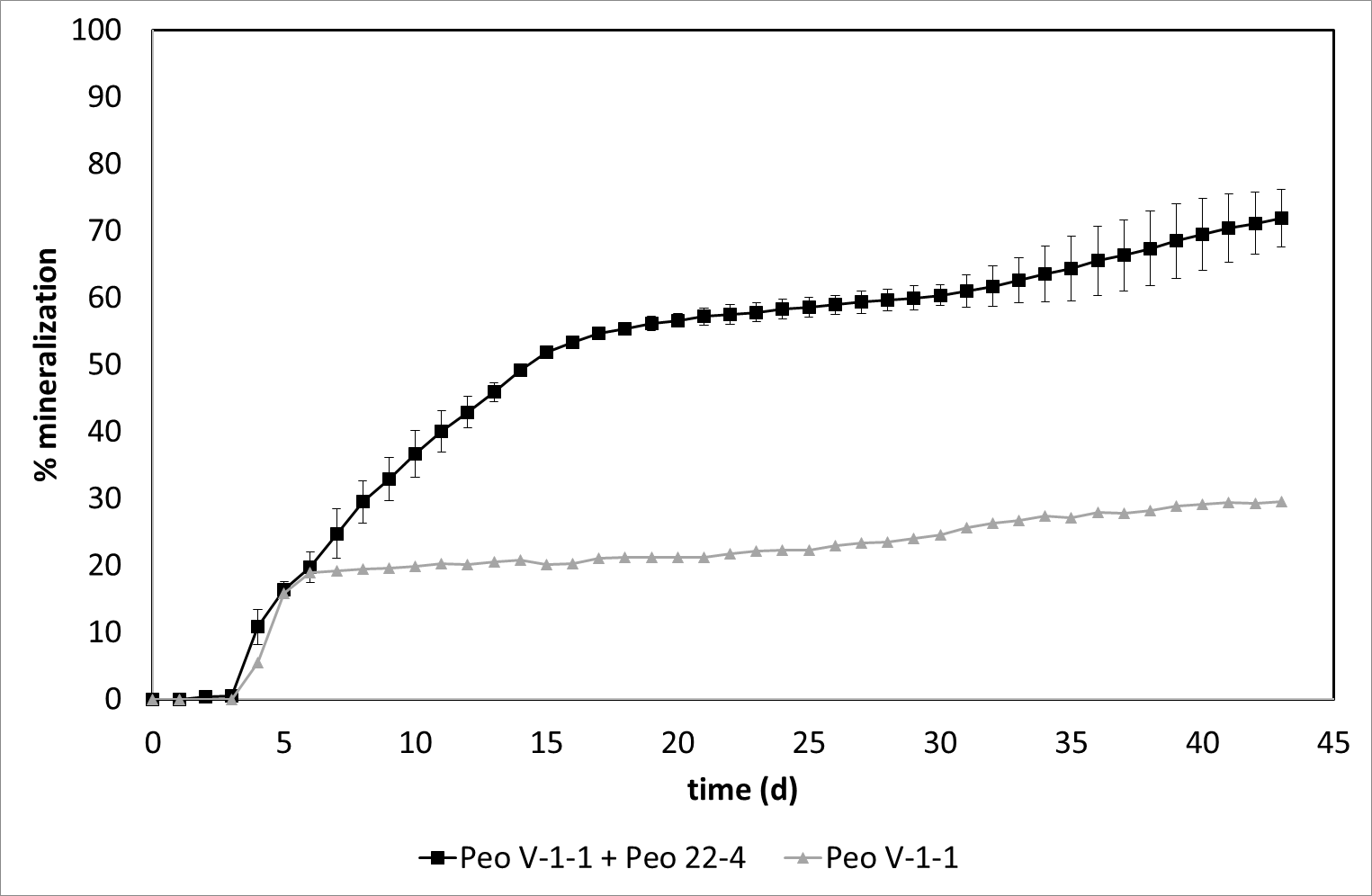


Figure S5. Biodegradation of Peonile by the bacterial isolates Peo-V-1-1 and a combination of Peo-V-1-1 and Peo-22-4. Measured with OxiTop heads under OECD 301F conditions (30 mg / L substrate). % Mineralization refers to % of theoretical oxygen demand consumed after subtraction of the oxygen consumption by cultures without substrate. Duplicate cultures for the consortium and singlicate for the strain Peo-V-1-1.


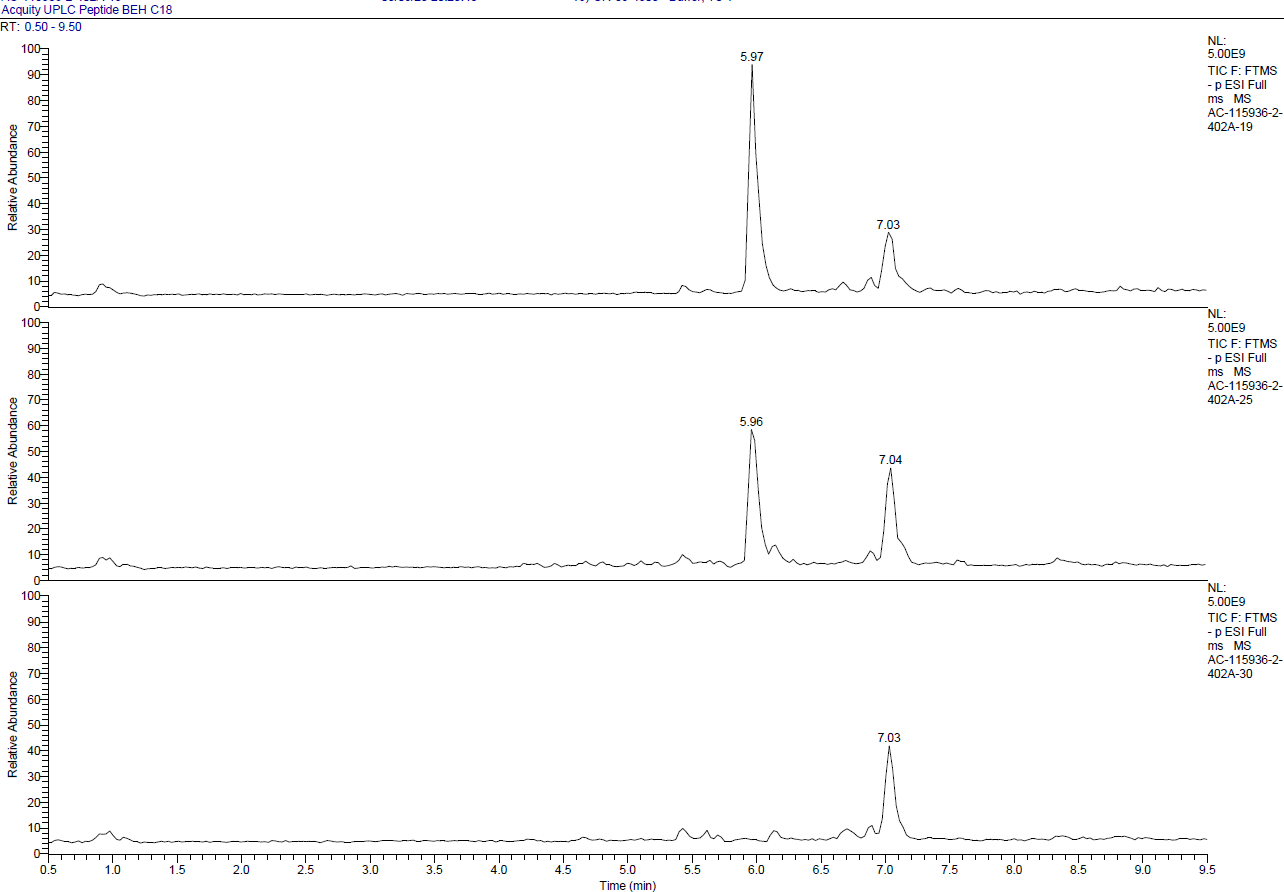


**a**

**b**

**c**

**c**

**c**

Figure S6. Incubation of 2CCHA with Peo-22-4 and analysis by LC-MS. Shown are total ion chromatograms for the total ion count (TIC) for (a) Substrate only at T = 0, (b) substrate incubated with Peo-22-4 for 5 min and (c) 60 min. 2CCHA (peak at 5.97 min) is quantitatively metabolized within 60 min without accumulation of significant amount of intermediates as observed by LC-MS. The peak at 7.03 min is a contaminant also present in the blank sample.
